# Supplementary material for: Format chain exchange (FORCE) for high-throughput generation of bispecific antibodies in combinatorial binder-format matrices
Source: Nat Commun. 2020 Oct 2;11:4974. doi: 10.1038/s41467-020-18477-7 (PMC7532213; doi:10.1038/s41467-020-18477-7)
Supplement: Supplementary file 1 — Supplementary Information [file 41467_2020_18477_MOESM1_ESM.pdf]

DATA SUPPLEMENT

Format Chain Exchange (FORCE) for high-throughput generation of bispecific antibodies in combinatorial binder-format matrices

Stefan Dengl[1], Klaus Mayer[1], Felix Bormann[1], Harald Duerr[1], Eike Hoffmann[1], Bianca Nussbaum[1], Michael Tischler[1], Martina Wagner[1], Andreas Kuglstatter[2], Lea Leibrock[2], Can Buldun[1], Guy Georges[1] and Ulrich Brinkmann[1]*

[1]Roche Pharma Research and Early Development (pRED), Large Molecule Research, Roche Innovation Center Munich, Penzberg, Germany

[2]Roche Pharma Research and Early Development (pRED), Structural Biology, Roche Innovation Center Basel, Basel, Switzerland

Supplementary Figure 1:	Yield and quality of dummy containing input molecules
Supplementary Figure 2:	SEC analyses demonstrate conversion of smaller sized educts to bsAb products
Supplementary Figure 3:	Differences in binding functionalities of bispecific binder-format combinations generated by FORCE
Supplementary Figure 4:	Four-parameter logistic (4PL) regression of ELISA Data
Supplementary Table 1:	Statistics for X-ray data processing and model refinement
Supplementary Table 2:	Robust and efficient process steps enable automation

Supplementary Figure 1

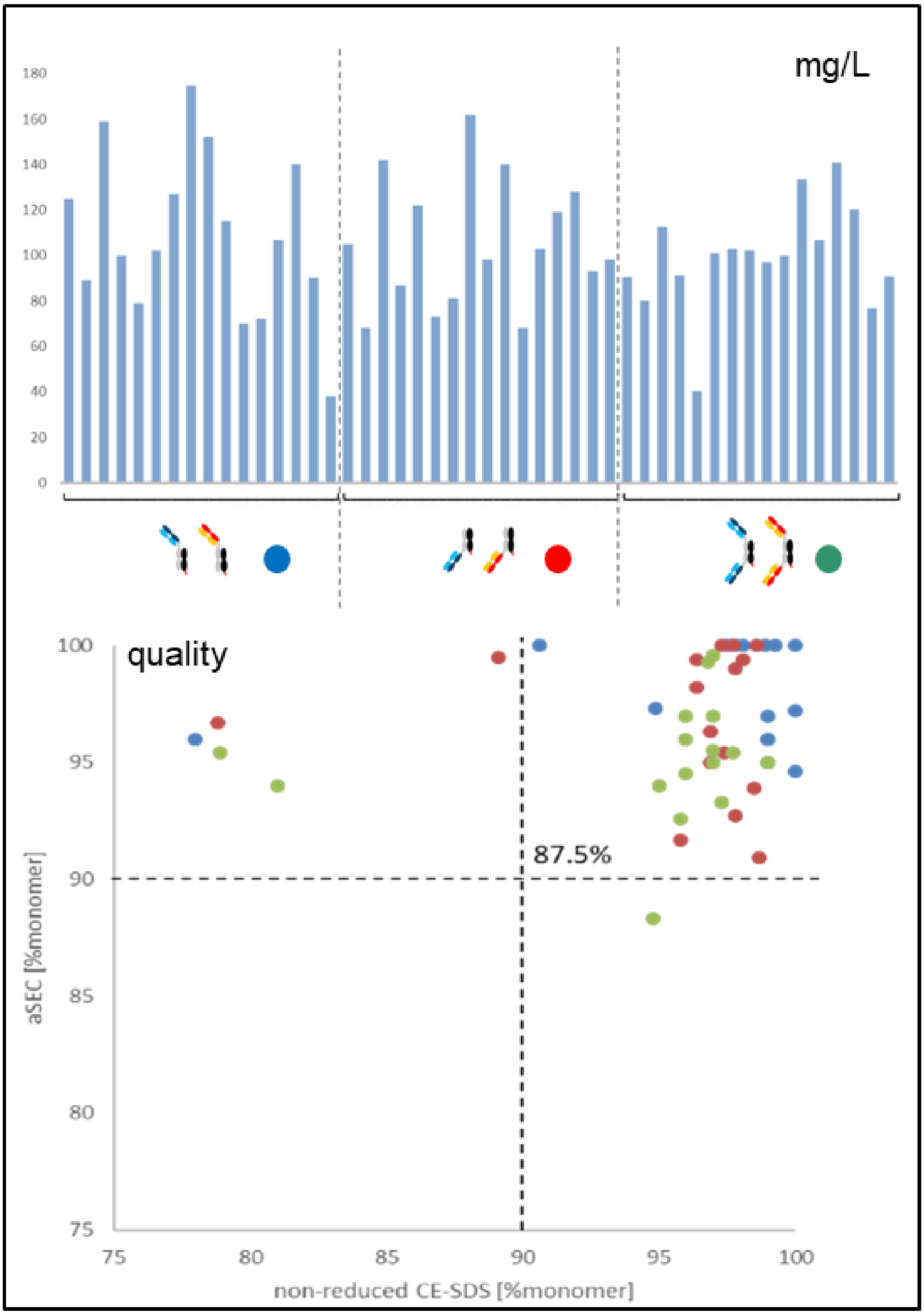

Supplementary Figure 1: Yield and quality of dummy containing input molecules
Biophysical properties and integrity of educts (or input molecules) were tested by analytical SEC, capillary electrophoresis (CE-SDS) and mass spectrometry. Here, exemplary data for 48 educts is shown. In general the Integrity and biochemical quality of the molecules is high with 87.5& of the molecules having analytical size exclusion and capillary SDS electrophoresis monomer peak ratios >90%. The few molecules that lie outside of this specification can still be used for subsequent bispec assembly, as the FORCE reaction includes an additional purification step after the exchange/assembly reaction.

Supplementary Figure 2

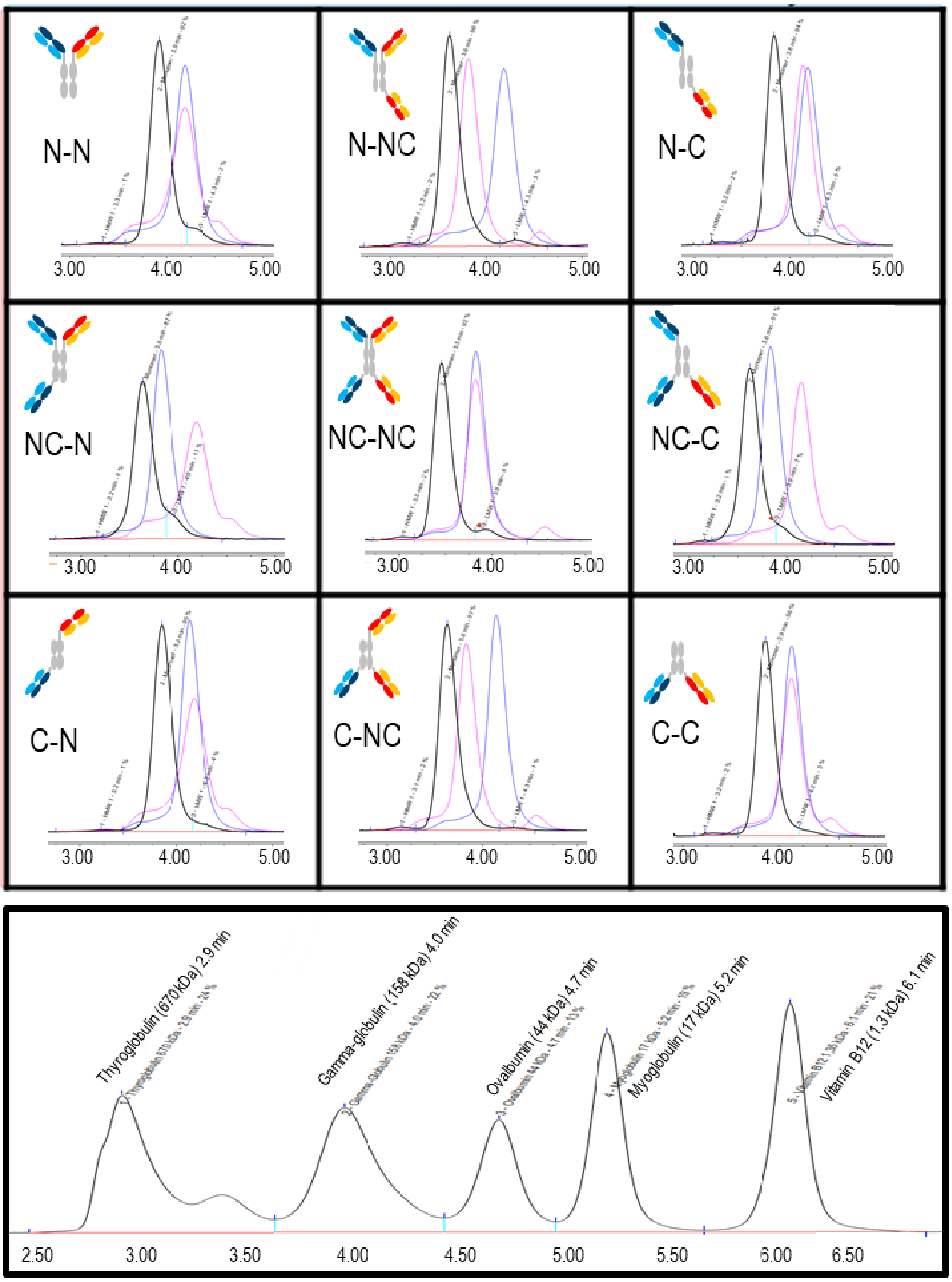

Supplementary Figure 2: SEC analyses demonstrate conversion of smaller sized educts to bsAb products.

The elution profile overlays show educts of the FORCE reaction in blue and red, products in black. N-N, NC-N, C-N, N-NC, NC-NC, C-NC, N-C, NC-C and C-C are the different formats generated via FORCE. Molecular weight standard markers (BioRad Gel Filtration Standard (Cat# 1511901) elute at 4 min (158 kDa gamma globulin) and 4.7 min (44 kDa ovalbumin) respectively. Labels attached in fine print to fractions are irrelevant yet accessible upon enlargement of the source data that are provided as part of the Source Data file.

Supplementary Figure 3

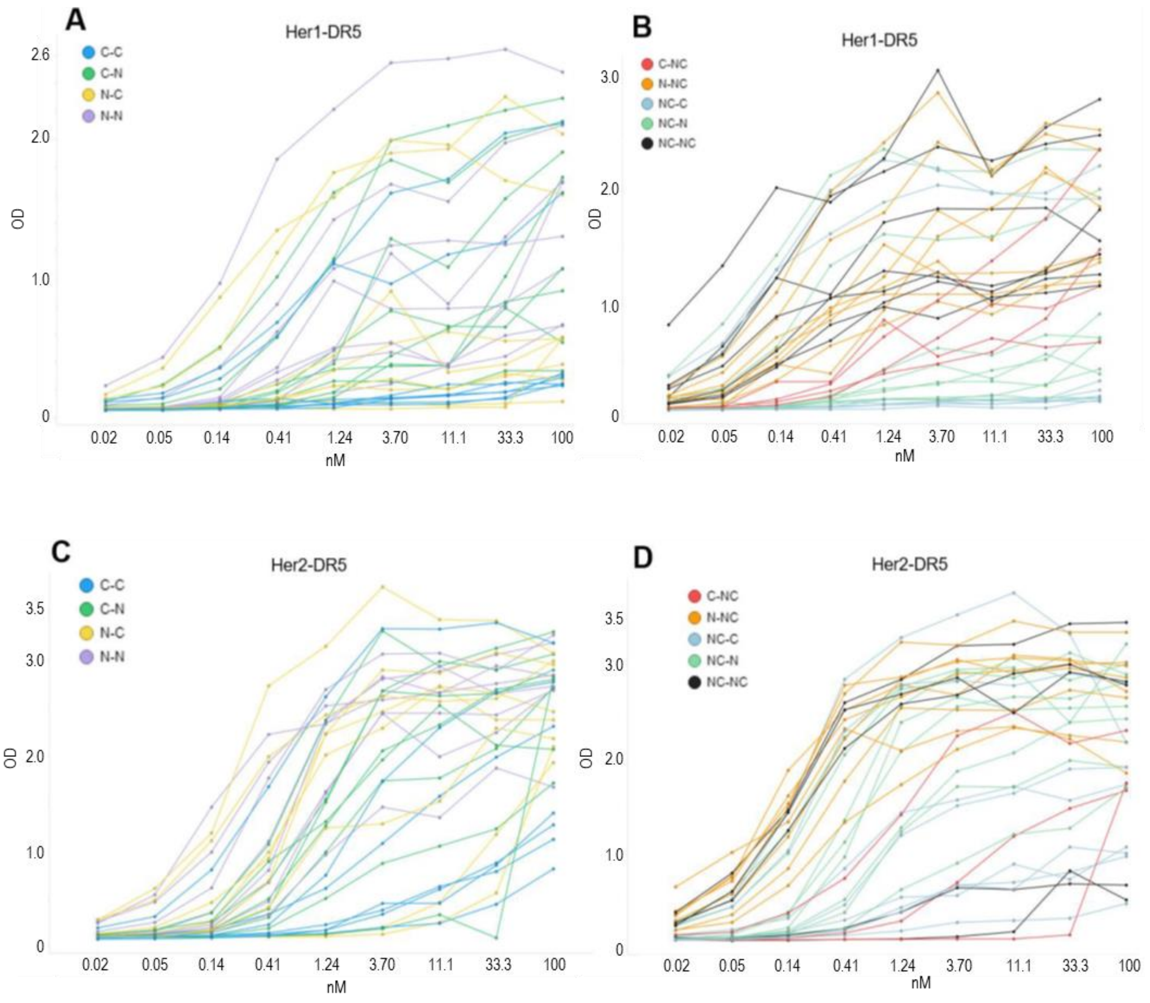

Supplementary Figure 3: Differences in binding functionalities of bispecific binder/format combinations generated by FORCE.

Bridging-ELISA dose response curves of A) 32 different monovalent and B) 35 different tri- and tetravalent formats containing the binding regions of Cetuximab and Imgatuzumab combined with anti-DR5 binding regions of Conatumumab, Drozitumab, KMTR2 and Tigatuzumab. Furthermore, dose-response curves of C) 31 different monovalent and D) 32 different tri- and tetravalent formats containing the binding regions of Trastuzumab and Pertuzumab combined with anti-DR5 binding regions of Conatumumab, Drozitumab, KMTR2 or Tigatuzumab, are shown. For these automated ELISA-based high throughput screens, individual samples were applied per protein and concentration (N=1), with each sample being part of a N=9 dose-response dilution group (N=9, 0.02-100nM). Determination of individual data points without replicates (N=1) is acceptable for high throughput screening approaches because (i) outliers are identified by gross deviations from N=9 dose-response data sets, (ii) statistics are thereafter performed based on N=9 dose-response area under the 4PL curve (Suppl. Figure 4) which reduces the effect of individual measurement outliers, and (iii) the main basis of subsequent data analysis are groups of samples with shared features which reduces the effect of individual sample preparation errors.

Supplementary Figure 4

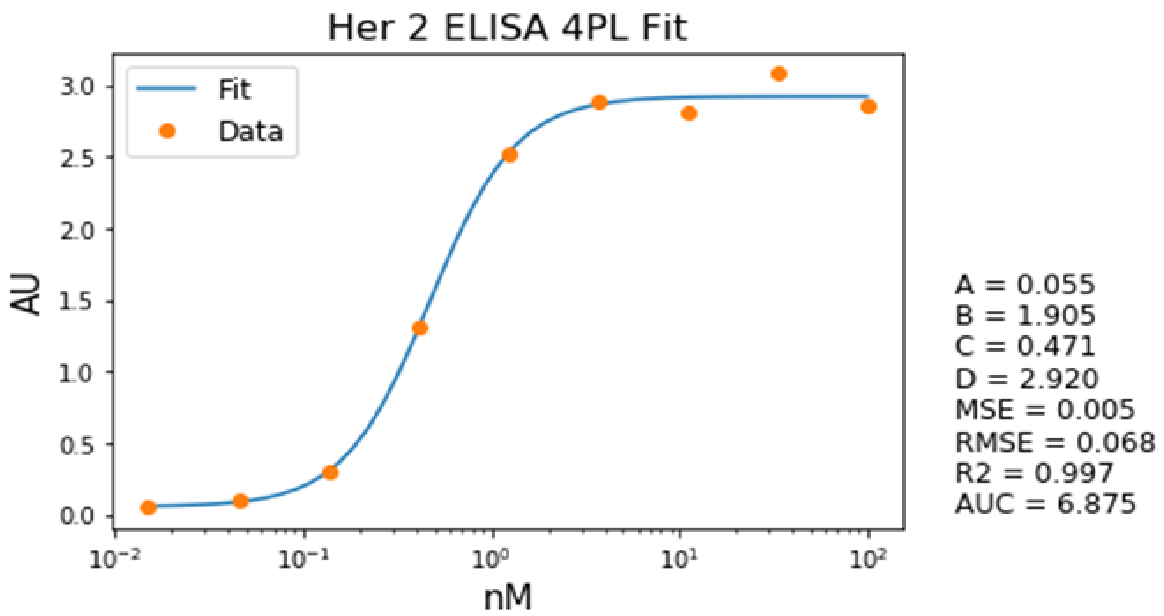

.

Supplementary Figure 4: Four-parameter logistic (4PL) regression of ELISA Data
Shown is one representative graph of 4PL-fit ELISA data (Her2 (Trastuzumab) -DR5 (Conatumumab) sample in NC-N format, one example of those listed in supplemental figure 3D). The graph depicts individual data points (N=1) of a high throughput screen. Individual data points in dose response curves can be applied to generate meaningful data in such screens because outliers are identified by gross deviations from N=9 dose-response data sets and statistics are thereafter performed based on N=9 dose-response (see Suppl.Fig.3 & Methods – Statistics & Reproducibility). A: bottom asymptote, B: Hill's slope, C: EC50, D: top asymptote, MSE: mean squared error, RMSE: root mean squared error, R2: R-squared, AUC: area under 4PL curve.

Supplementary Table 1: Statistics for X-ray data processing and model refinement

**PDB Accession Number**	**6YTB**	**6YSC**	**6YT7**
**Data Processing**[a]			
Space Group	$P2_12_12_1$	$P2_12_12_1$	$P2_12_12_1$
Unit cell axes [Å]	49.1/75.2/149.5	49.4/80.4/141.8	49.0/75.1/149.4
Resolution limits [Å]	74.74-1.66 (1.73-1.66)	69.92-2.05 (2.21-2.05)	74.83-1.55 (1.69-1.55)
Completeness, ellipsoidal [%]	95.1 (51.5)	94.8 (68.3)	95.4 (61.8)
R_{pim}	0.024 (0.568)	0.024 (0.423)	0.020 (0.542)
$I/\sigma(I)$	16.2 (1.3)	17.4 (1.5)	23.1 (1.6)
CC1/2	1.00 (0.57)	1.00 (0.64)	1.00 (0.70)
Multiplicity	6.6 (6.6)	6.5 (6.9)	6.6 (6.9)
**Refinement**			
No. reflections	61292	24877	64377
R/R_{free} [%]	21.6/24.9	23.3/29.3	23.4/27.3
Rmsd bond length [Å]	0.011	0.009	0.012
Rmsd bond angles [°]	1.52	1.95	2.39
Ramachandran favored [%]	97	95	96
Ramachandran outliers [%]	0.97	0.25	1.21
B factor, overall [$Å^2$]	37.9	47.9	36.1
B factor, protein [$Å^2$]	37.2	47.2	35.5
B factor, water [$Å^2$]	37.1	37.6	34.6

[a] Number in parenthesis are values for the highest of ten resolution shells.

Supplementary Table 2: Robust and efficient process steps enable automation

process step	operation	hardware	products
expression of educts	30ml HEK Expi in 6-well plates with supernatants transferred to 24 well plates	customized system	cleared supernatants
purification of educts: capturing	small scale ProtA, 600 µl	Tecan freedom Evo, Atoll robocolumn station and high volume dilutors	2.5-4 mg educts
purification of educts: SEC	Superdex 200, 10/30	Dionex HPLC, interlaced preparative SEC	2-3 mg educts
exchange reaction	Mixing of educts, TCEP addition, incubation, 96 well	Tecan freedom Evo	360-500 µl per reaction samples (depending on MW of educts)
bsAb purification & characterization	adsorbtion of tagged proteins, flow-through mode; CE-SDS and analytical SEC	Tecan freedom Evo; Atoll robocolumn station; GX II CE-SDS; Dionex HPLC	purified bsAbs
assessment of bsAb functionality	384 well biochem or cellular assays	CybiWell liquid handler	bsAb efficacy ranking, candidates

The custom-designed transfection and purification systems combine different commercially available modules (incl. liquid handling / transport robots, sterile boxing, incubator, harvesting centrifuge, CO2-incubator, media/buffer/reagent reservoir stations). These were assembled into a transfection robot with custom-designed interfaces according to our specifications by a company specialized in lab-automation. The transient HEK expression in 30ml scale is performed on this system in 6-deepwell microplates (#CR1406, EnzyScreen, Netherlands) followed by centrifugation. Thereafter, samples are transferred into 24-deepwell microplates (#360080, Porvair Sciences, UK). Thus, the 30 ml transient expression supernatant is distributed to 3 wells on the 24-deepwell microplates for subsequent purification.
