# Supplementary material for: Format chain exchange (FORCE) for high-throughput generation of bispecific antibodies in combinatorial binder-format matrices
Source: Nat Commun. 2020 Oct 2;11:4974. doi: 10.1038/s41467-020-18477-7 (PMC7532213; doi:10.1038/s41467-020-18477-7)
Supplement: Supplementary file 2 — Reporting Summary [file 41467_2020_18477_MOESM2_ESM.pdf]

nature research

Corresponding author(s): Ulrich Brinkmann

Last updated by author(s): Aug 13, 2020

# Reporting Summary

Nature Research wishes to improve the reproducibility of the work that we publish. This form provides structure for consistency and transparency in reporting. For further information on Nature Research policies, see our Editorial Policies and the Editorial Policy Checklist.

## Statistics

For all statistical analyses, confirm that the following items are present in the figure legend, table legend, main text, or Methods section.

| n/a | Confirmed | |
|---|---|---|
| ☐ | ☒ | The exact sample size (*n*) for each experimental group/condition, given as a discrete number and unit of measurement |
| ☐ | ☒ | A statement on whether measurements were taken from distinct samples or whether the same sample was measured repeatedly |
| ☐ | ☒ | The statistical test(s) used AND whether they are one- or two-sided<br>*Only common tests should be described solely by name; describe more complex techniques in the Methods section.* |
| ☐ | ☒ | A description of all covariates tested |
| ☐ | ☒ | A description of any assumptions or corrections, such as tests of normality and adjustment for multiple comparisons |
| ☐ | ☒ | A full description of the statistical parameters including central tendency (e.g. means) or other basic estimates (e.g. regression coefficient) AND variation (e.g. standard deviation) or associated estimates of uncertainty (e.g. confidence intervals) |
| ☐ | ☒ | For null hypothesis testing, the test statistic (e.g. *F*, *t*, *r*) with confidence intervals, effect sizes, degrees of freedom and *P* value noted<br>*Give P values as exact values whenever suitable.* |
| ☒ | ☐ | For Bayesian analysis, information on the choice of priors and Markov chain Monte Carlo settings |
| ☒ | ☐ | For hierarchical and complex designs, identification of the appropriate level for tests and full reporting of outcomes |
| ☒ | ☐ | Estimates of effect sizes (e.g. Cohen's *d*, Pearson's *r*), indicating how they were calculated |

*Our web collection on statistics for biologists contains articles on many of the points above.*

## Software and code

Policy information about availability of computer code

| | |
|---|---|
| Data collection | Data were assembled and processed by Spotfire (Spotfire Analyst 7.11.0 LTS, © 2007 2017 TIBCO Software Inc, Build date: 5/29/2018) and Microsoft Excel 2016 32bit. |
| Data analysis | Spotfire (Spotfire Analyst 7.11.0 LTS, © 2007 2017 TIBCO Software Inc, Build date: 5/29/2018) for early data compilation & inspection. Calculations in Python (3.6) using NumPy (1.18.4), Pandas (1.0.3) and SciPy (1.2.3) and plots visualized using Seaborn (0.9.1). The structures were determined by molecular replacement with the program PHENIX (1.18.2, https://www.phenix-online.org) and refined with the programs REFMAC5 (https://www2.mrc-lmb.cam.ac.uk/groups/murshudov/) and phenix.refine (1.18.2, https://www.phenix-online.org). Represention of pdb structures via DiscoveryStudio: Dassault Systèmes BIOVIA, Discovery Studio Modeling Environment, Release 2017, San Diego: Dassault Systèmes, 2016. |

For manuscripts utilizing custom algorithms or software that are central to the research but not yet described in published literature, software must be made available to editors and reviewers. We strongly encourage code deposition in a community repository (e.g. GitHub). See the Nature Research guidelines for submitting code & software for further information.

## Data

Policy information about availability of data

All manuscripts must include a data availability statement. This statement should provide the following information, where applicable:

- Accession codes, unique identifiers, or web links for publicly available datasets
- A list of figures that have associated raw data
- A description of any restrictions on data availability

Data availibility statement: All mutations are described by position and identity and confirmed by Xray structure determination. The structures that we generated as part of this study are deposited in PDB, ID’s 6YTB, 6YT7, 6YSC. Raw data that form the basis of Her1/2-DR5 bsAb experiments are provided in Supplementary Figure

3, additional raw data in the source data file.

# Field-specific reporting

Please select the one below that is the best fit for your research. If you are not sure, read the appropriate sections before making your selection.

☒ Life sciences ☐ Behavioural & social sciences ☐ Ecological, evolutionary & environmental sciences

For a reference copy of the document with all sections, see nature.com/documents/nr-reporting-summary-flat.pdf

# Life sciences study design

All studies must disclose on these points even when the disclosure is negative.

| | |
|---|---|
| Sample size | Multiple samples & repeated experiments for platform design, set-up and validation, single samples in high-throughput screens, clustered to dose-respones groups and structural multi-sample 'families' for determination of rules.<br>Sample sizes and justifications are provided in the figure legends and explained in Methods - Statistics & Reproducibility. |
| Data exclusions | all available data were included, no exclusion |
| Replication | Throughout reproducible results for experimental repetitions (platform design, set-up and validation), single samples in high-throughput screens, clustered to structural multi-sample 'families' for determination of rules. Sample sizes and justifications are provided in the figure legends and explained in Methods - Statistics & Reproducibility. |
| Randomization | no randomization - randomization is not applicable for this study because it covers rational structural protein design & biochemical characterization |
| Blinding | no blinding - blinding not applicable for this study because it covers rational structural protein design & biochemical characterization |

# Reporting for specific materials, systems and methods

We require information from authors about some types of materials, experimental systems and methods used in many studies. Here, indicate whether each material, system or method listed is relevant to your study. If you are not sure if a list item applies to your research, read the appropriate section before selecting a response.

## Materials & experimental systems

| n/a | Involved in the study | |
|---|---|---|
| ☐ | ☒ | Antibodies |
| ☐ | ☒ | Eukaryotic cell lines |
| ☒ | ☐ | Palaeontology and archaeology |
| ☒ | ☐ | Animals and other organisms |
| ☒ | ☐ | Human research participants |
| ☒ | ☐ | Clinical data |
| ☒ | ☐ | Dual use research of concern |

## Methods

| n/a | Involved in the study | |
|---|---|---|
| ☒ | ☐ | ChIP-seq |
| ☒ | ☐ | Flow cytometry |
| ☒ | ☐ | MRI-based neuroimaging |

## Antibodies

| | |
|---|---|
| Antibodies used | The study does not apply commercial antibodies / antibody reagents. Instead, all antibodies were recombinantly produced as part of this study. They are recombinant derivatives of Conatumumab, Drozitumab, Tigatuzumab, KMTR2, Cetuximab, Imgatuzumab, Pertuzumab, Trastuzumab, based on sequences described in the cited references. |
| Validation | The study does not apply commercial antibodies. All antibody derivatives that were recombinantly produced as part of this study were sequence-confirmed. |

## Eukaryotic cell lines

Policy information about cell lines

| | |
|---|---|
| Cell line source(s) | Human embryonic kidney 293-F cells (FreeStyle™ 293 system, ThermoFisher) and HEK Expi (ThermoFisher). |
| Authentication | cell lines were commercially obtained (ThermoFisher) and used without further authentication, except for confirmation of expected functionality as expression hosts for secretion of recombinant antibodies. |
| Mycoplasma contamination | negative (in-house tested) |

| | |
|---|---|
| Commonly misidentified lines<br>(See ICLAC register) | none |
